# Male Gender Expressivity and Diagnosis and Treatment of Cardiovascular Disease Risks in Men

**DOI:** 10.1001/jamanetworkopen.2024.41281

**Published:** 2024-10-25

**Authors:** Nathaniel J. Glasser, Jacob C. Jameson, Elbert S. Huang, Ian M. Kronish, Stacy Tessler Lindau, Monica E. Peek, Elizabeth L. Tung, Harold A. Pollack

**Affiliations:** 1 Section of General Internal Medicine, University of Chicago, Chicago, Illinois; 2 Center for Health Decision Science, Harvard T. H. Chan School of Public Health, Boston, Massachusetts; 3 Center for Chronic Disease Research and Policy, University of Chicago, Chicago, Illinois; 4 Center for Behavioral Cardiovascular Health, Columbia University Irving Medical Center, New York, New York; 5 Department of Obstetrics & Gynecology, University of Chicago, Chicago, Illinois; 6 Department of Medicine-Geriatrics and Palliative Medicine, University of Chicago, Chicago, Illinois; 7 MacLean Center for Medical Ethics, University of Chicago, Chicago, Illinois; 8 Center for the Study of Race, Politics, and Culture, University of Chicago, Chicago, Illinois; 9 Center for Center for Diabetes Translation Research, University of Chicago, Chicago, Illinois; 10 Crown Family School of Social Work, Policy and Practice, University of Chicago, Chicago, Illinois; 11 University of Chicago Health Lab, University of Chicago, Chicago, Illinois; 12 Department of Public Health Sciences, Biological Sciences Division, University of Chicago, Chicago, Illinois

## Abstract

**Importance:**

Male gender expressivity (MGE), which reflects prevalent sociocultural pressures to convey masculinity, has been associated with health. Yet, little is known about associations of MGE with the diagnosis and treatment of modifiable cardiovascular disease (CVD) risks.

**Objective:**

To investigate associations of MGE with modifiable CVD risk diagnoses and treatment in men.

**Design, Setting, and Participants:**

This population-based cohort study included data from waves I (1994-1995), IV (2008-2009), and V (2016-2018) of the US National Longitudinal Study of Adolescent to Adult Health (Add Health). Participants were male adolescents (age 12-18 years) followed up longitudinally through younger adulthood (age 24-32 years) and adulthood (age 32-42 years). Data were analyzed from January 5, 2023, to August 28, 2024.

**Exposure:**

Male gender expressivity was quantified in adolescence and younger adulthood using an empirically-derived and validated measurement technique that incorporates participants' responses to existing Add Health survey items to capture how similarly participants behave to same-gendered peers.

**Main Outcomes and Measures:**

Outcomes included self-reported diagnoses of CVD risk conditions (hypertension, diabetes, or hyperlipidemia) in adult men with elevated blood pressure, hemoglobin A_1c_, or non–high-density lipoprotein cholesterol levels, and self-reported treatment with antihypertensive, hypoglycemic, or lipid-lowering medications in adults reporting hypertension, diabetes, or hyperlipidemia. Multivariable regression was used to examine associations of adolescent and younger adult MGE with adult CVD risk diagnoses and treatment, adjusting for sociodemographic covariates.

**Results:**

Among 4230 eligible male participants, most were non-Hispanic White (2711 [64%]) and privately insured (3338 [80%]). Their mean (SD) age was 16.14 (1.81) years in adolescence, 29.02 (1.84) years in younger adulthood, and 38.10 (1.95) years in adulthood. Compared with participants whose younger adult MGE was below average, those with higher younger adult MGE were overall less likely to report hypertension (22% vs 26%; *P* < .001), diabetes (5% vs 8%; *P* < .001), and hyperlipidemia (19% vs 24%; *P* < .001) diagnoses and diabetes treatment (3% vs 5%; *P* = .02) as adults. In multivariable models, every SD increase in adolescent MGE was associated with lower probabilities of adult hypertension treatment (MGE,−0.11; 95% CI, −0.16 to −0.6) and diabetes diagnoses (MGE, −0.15; 95% CI, −0.27 to −0.03). Higher younger adult MGE was associated with lower probabilities of adult hypertension diagnoses (MGE, −0.04; 95% CI, −0.07 to −0.01), hypertension treatment (MGE, −0.07; 95% CI, −0.13 to −0.01), and diabetes treatment (MGE, −0.10; 95% CI, −0.20 to −0.01). Adolescent and younger adult MGE outcomes were not associated with other adult CVD outcomes.

**Conclusions and Relevance:**

In this cohort study of US males, higher adolescent and younger adult MGE was associated with lower adult hypertension and diabetes diagnoses and treatment. These findings suggest that males with high MGE may bear distinctive risks and correspondingly benefit from tailored public health efforts to prevent downstream CVD.

## Key Points

**Question:**

What is the association of male gender expressivity (MGE)—a measure reflecting sociocultural pressures to convey male gender identity—with the diagnosis and treatment of modifiable cardiovascular disease risks?

**Findings:**

This cohort study involving 4230 male participants found that every SD increase in participants’ adolescent MGE was associated with an 11-percentage point lower probability of adult hypertension treatment and 15-percentage point lower probability of adult diabetes diagnosis. Higher younger adult MGE was associated with lower adult probabilities of hypertension diagnosis, hypertension treatment, and diabetes treatment.

**Meaning:**

The findings of this study suggest that sociocultural pressures to convey male gender may be associated with suboptimal cardiovascular disease outcomes through decreased risk diagnosis and treatment.

## Teaser

This cohort study examines the association between male gender expressivity--a measure reflecting sociocultural pressures to convey male gender identity--during adolescence and adulthood and diagnosis and treatment of cardiovascular disease (CVD) risks in men.

## Introduction

Prevalent sociocultural pressures (eg, narratives, norms, values, and expectations) to convey male gender identity have been shown to shape boys’ and men’s behaviors, preferences, and beliefs.^1,2,3,4^ These pressures frequently encourage displays of self-reliance, emotional control, and strength while discouraging help-seeking, vulnerability, or weakness.^1,2,3,4,5,6,7^ The degree to which boys and men adopt behaviors similar to those of same-gendered peers (and different from those of other genders)—their male gender expressivity (MGE)—has been understood and measured as a proxy for the association between these pressures and their behaviors and outlook.^8,9,10^ Increasingly, MGE and related measures have been linked to health behaviors,^11^ including substance use^10,11,12,13^ and COVID-19 prevention.^14^ Previous analyses have observed that individuals’ MGE at developmental stages as early as adolescence might forecast downstream outcomes, such as tobacco use and weight control.^10,11,13,15,16^

Cardiovascular disease (CVD), a leading cause of morbidity and mortality in the US and globally,^17,18,19,20^ results in well-established sex- and gender-based health disparities.^21,22,23,24,25,26^ Yet, except for several small, primarily qualitative inquiries, we know of no studies examining associations between MGE and CVD outcomes in a nationally representative US sample.^4,27,28,29^ The qualitative studies provide evidence of how various context-specific pressures to convey male gender appear to influence help-seeking for CVD-related symptoms and diagnoses among men, often leading to suppressed help-seeking efforts.^4,27,28^

Further evidence suggests that even in younger adults, including those with only borderline evidence of CVD risk factors, such as hypertension, diabetes, and hyperlipidemia, the presence of these risk factors may be associated with increased downstream CVD-related morbidity and mortality, underscoring the clinical and public health importance of early recognition and treatment.^30,31,32,33,34,35,36,37,38,39,40,41^ Many guidelines resultingly now recommend universal screening for hypertension and hyperlipidemia in children and adolescents^42,43^ in addition to broadly targeted screening for hypertension, diabetes, and hyperlipidemia in adults.^44,45,46,47^ Despite this emphasis on early detection, evidence suggests that up to 75% of younger adults with uncontrolled CVD risk factors are unaware they have these conditions.^40,41^ Prior studies suggest that younger age, lack of insurance, and no regular source of preventive health care are generally associated with lower CVD risk awareness.^40,41,48,49,50^

We used data from the National Longitudinal Study of Adolescent to Adult Health (Add Health) to investigate associations of adolescent and younger adult MGE with adult diagnoses and treatment of modifiable CVD risks, namely hypertension, diabetes, and hyperlipidemia. Existing, primarily qualitative, evidence suggests that boys and men experience especially strong social pressures to portray gender-congruent behaviors that emphasize dominance and deny vulnerability,^3,4,6,51^ including through the avoidance of preventive health care and rejection of recommended medical therapies.^7,14,52,53,54^ We thus hypothesized that increased MGE is associated with lower diagnoses and treatment of CVD risks.

## Methods

This longitudinal cohort study used data from waves I (1994-1995), IV (2008-2009), and V (2016-2018) of Add Health. Participants were adolescents (age 12-18 years) in wave I, younger adults (age 24-32 years) in wave IV, and adults (age 32-42 years) in wave V. Wave I participants (n = 20 745; 10 263 male) comprised a randomly selected, nationally representative probability sample of US adolescents. The sample included in the present analysis was restricted to respondents followed up through waves IV (n = 15 197; 7341 male) and V (n = 12 300; 5324 males) who participated and identified as male in all 3 waves (n = 4230) (eFigure in Supplement 1). A smaller subset that participated in biomeasure collection in wave V (n = 5381; 2132 males) was included in analyses involving biomeasure and medication use data. Add Health participants provided written informed consent for participation in all aspects of Add Health in accordance with the University of North Carolina School of Public Health Institutional Review Board. This secondary analysis of previously collected Add Health data was approved by the University of Chicago Institutional Review Board. The study followed the Strengthening the Reporting of Observational Studies in Epidemiology (STROBE) reporting guideline^55^

To quantify MGE, we used a reliable valid measure developed from Add Health data and applied in multiple prior analyses.^9,10,11,13^ The measure uses participants’ responses to the 25 wave I and 22 wave IV Add Health survey items answered most differently by female vs male participants (eTable 1 in Supplement 1). Participants’ responses were then used in logistic regression models predicting their reported gender. Male participants’ scores thus allowed us to quantify their MGE by capturing how similarly they responded to same-gendered peers on survey items that elicited the largest gender-based differences in responses. Predicted probabilities were standardized using *z* scores across male participants in each wave such that 0 represented the mean, +1 represented 1 SD above, and so on. Empirically derived, the measure does not project contemporary norms onto noncontemporary samples. Instead, participants’ responses themselves are used to construct the measure.^10^

Given prior findings that MGE typically develops in adolescence but evolves into younger adulthood, separate MGE scores were calculated for adolescence and younger adulthood.^9,11,56,57^ We also measured participants’ adolescent to younger adult MGE change as the difference between adolescent and younger adult MGE *z* scores.

Primary dependent variables were constructed from adult (wave V) data to allow greater power as CVD risk becomes increasingly detectable with age.^58,59^ Binary variables were based on yes and no responses to survey items, asking “whether a doctor, nurse, or other health care provider ever told you that you have or had [high blood pressure or hypertension, high blood sugar or diabetes, high blood cholesterol, triglycerides, lipids, or hyperlipidemia].” Yes responses indicated diagnoses of hypertension, diabetes, or hyperlipidemia.

Consistent with current American Heart Association/American College of Cardiology Task Force guidance on hypertension classification,^60^ elevated blood pressure (BP) was defined as systolic BP exceeding 130 mm Hg or diastolic BP exceeding 80 mm Hg based on the average of 3 measurements obtained using factory-calibrated oscillometric BP monitors (Microlife Corp).^61^ Consistent with the American Diabetes Association diabetes classification,^62^ an elevated hemoglobin A_1c_ (HbA_1c_) level was defined as 6.5% or greater (to convert to proportion of total hemoglobin, multiply by 0.01) on a venous blood sample.^63^ Hyperlipidemia was defined as serum non–high-density lipoprotein cholesterol (HDL-C) 190 mg/dL (to convert to millimoles per liter, multiply by 0.259) on venous blood samples, consistent with 2019 American Heart Association/American College of Cardiology guidance.^47,64,65^ The non–HDL-C measure was chosen given evidence of its utility in predicting CVD mortality.^65^

Finally, treatment was assessed by participants’ self-report of all medications used during the 4 weeks before biomeasure collection. Medications were categorized based on classifications listed in Micromedex and Lexicomp as antihypertensive, hypoglycemic, and lipid-lowering.^66^ Treatment was defined as self-reporting at least 1 medication for a corresponding risk category.

Participant-level covariates included self-reported adult race and ethnicity (participants categorized as “other” included those who indicated “some other race or origin” and those who indicated multiple races but did not indicate a specific race), educational attainment, and health insurance status on close-coded survey questions, and a composite measure of adolescent socioeconomic origin.^67^ Neighborhood-level covariates included a composite measure of adolescent neighborhood socioeconomic disadvantage.^67^ Individual- and neighborhood-level sociodemographic covariates (including race and ethnicity) were included to account for possible sociodemographic differences in the association between MGE and CVD prevention efforts.

### Statistical Analysis

Data were analyzed from January 5, 2023, to August 28, 2024. Descriptive statistics were calculated for participant- and neighborhood-level characteristics. Logistic regression was used to examine associations of adolescent, younger adult, and adolescent-to-younger-adult MGE change, controlling for adolescent MGE, with adult CVD risk outcomes. Average marginal effects (dy/dx) were then calculated, which illustrate associations of differences in MGE at each developmental stage with differences in the predicted probabilities of adult CVD outcomes.

In model 1, we evaluated associations of adolescent, younger adult, and adolescent-to-younger adult MGE changes with adult hypertension, diabetes, and hyperlipidemia diagnoses. This model included an interaction term between MGE and relevant biomeasure levels (ie, models testing associations of MGE with hypertension diagnoses included an interaction with BP, diabetes with HbA_1c_, and hyperlipidemia with non–HDL-C). This interaction term allowed us to test associations of MGE with adult CVD risk diagnoses, specifically among men with biomeasure evidence of the disease. Model 2 assessed associations of MGE with treatment among adult men who reported relevant diagnoses (eg, evaluation of antihypertensive use included an interaction term between MGE and self-reported hypertension diagnoses). Model 3 evaluated associations of MGE with adult biomeasure levels (BP, HbA_1c_, and non–HDL-C). This model adjusted for relevant medication use (eg, regressions modeling associations with BP adjusted for antihypertensive use). All models adjusted for individual- and neighborhood-level sociodemographic covariates. Sensitivity analyses were conducted, assessing BP at a higher threshold (systolic BP≥140 mm Hg or diastolic BP≥90 mm Hg) and associations of MGE with treatment adjusting for biomeasure level.

Add Health sampling weights were incorporated into regression models to account for the complex survey design of Add Health, which included an unequal probability of selection, differential survey-item nonresponse, and missing surveys (ie, participants not interviewed during a particular wave). Further analysis of item missingness is provided in eTable 2 in Supplement 1 and comparison of participants with complete vs missing covariate data in eTable 3 in Supplement 1.

Significance levels were set at *P* < .05 for 2-tailed tests. Analyses were performed using Stata SE, version 17 (StataCorp LLC) and replicated using R Programming Language, version 4.3.3 (R Foundation for Statistical Computing).

## Results

Overall, 4230 male participants were included in the study (Table 1). Their mean (SD) age was 16.14 (1.81) years in adolescence, 29.02 (1.84) years in younger adulthood, and 38.10 (1.95) years in adulthood. Participants self-identified as Asian American or Pacific Islander (298 [7%]), Hispanic (487 [12%]), non-Hispanic Black (668 [16%]), non-Hispanic White (2711 [64%]), or multiple race and/or ethnicity or other (54 [1%]). Most participants were privately insured (3338 [80%]). Participants whose younger adult MGE was above average, compared with those whose MGE was below average, were more likely to be White (1692 [67%] vs 1019 [60%]; *P* < .001) and report a primarily military source of insurance (eg, TRICARE; 139 [6%] vs 23 [1%]; *P* < .001), but less likely to have received a college degree (887 [35%] vs 720 [42%]; *P* < .001). They were also significantly less likely to report diagnoses of hypertension (546 [22%] vs 441 [26%]; *P* < .001), diabetes (124 [5%] vs 143 [8%]; *P* < .001), and hyperlipidemia (488 [19%] vs 406 [24%]; *P* < .001), although only increased HbA_1c_ (ie, not BP or non–HDL-C) was significantly less prevalent in this group (39 [4%] vs 48 [7%]; *P* = .008). Overall, the prevalence of increased CVD risk factor biomeasure levels was 64% for BP, 5% for HbA_1c_, and 10% for non–HDL-C.

**Table 1.** Demographic Characteristics of Males Included in the Analytic Sample

Sample characteristics (N = 4230)	No. (%)			*P* value^a^
	Total	MGE in younger adulthood		
		Below average (n = 1698)	Above average (n = 2532)	
Race and ethnicity				
Asian American or Pacific Islander	298 (7)	127 (8)	171 (7)	<.001
Hispanic	487 (12)	264 (16)	223 (9)	
Non-Hispanic Black	668 (16)	269 (16)	399 (16)	
Non-Hispanic White	2711 (64)	1019 (60)	1692 (67)	
Multiple race and/or ethnicity or other^b^	54 (1)	14 (1)	40 (2)	
Educational attainment				
Some high school or less	168 (4)	73 (4)	95 (4)	<.001
High school diploma or GED	722 (17)	264 (16)	458 (18)	
Some college or technical/associate degree	1730 (41)	641 (38)	1089 (43)	
College degree or more	1607 (38)	720 (42)	887 (35)	
Insurance status				
Private	3338 (80)	1393 (83)	1945 (78)	<.001
Medicaid or Medicare	308 (7)	127 (8)	181 (7)	
Other governmental insurance^c^	162 (4)	23 (1)	139 (6)	
Uninsured	389 (9)	144 (9)	245 (10)	
Adolescent socioeconomic disadvantage, mean (range)	0.51 (−4.67 to 3.51)	0.51 (−4.48 to 3.12)	0.49 (−4.67 to 3.51)	.30
Adolescent neighborhood disadvantage, mean (range)	23 (5 to 50)	23 (5 to 50)	23 (5 to 50)	.14
Self-report of hypertension diagnosis^d^				
No	3223 (77)	1249 (74)	1974 (78)	<.001
Yes	987 (23)	441 (26)	546 (22)	
BP, mm Hg^e^				
Normal	651 (36)	274 (37)	377 (35)	.41
Increased	1168 (64)	475 (63)	693 (65)	
Antihypertensive medication use^f^				
No	1589 (87)	641 (85)	948 (88)	.13
Yes	243 (13)	112 (15)	131 (12)	
Self-report of diabetes diagnosis^d^				
No	3952 (94)	1551 (92)	2401 (95)	<.001
Yes	267 (6)	143 (8)	124 (5)	
HbA_1c_, %				
<6.5	1564 (95)	636 (93)	928 (96)	.008
≥6.5	87 (5)	48 (7)	39 (4)	
Hypoglycemic medication use^f^				
No	1758 (96)	713 (95)	1045 (97)	.02
Yes	73 (4)	40 (5)	33 (3)	
Self-report of hyperlipidemia diagnosis^d^				
No	3311 (79)	1284 (76)	2027 (81)	<.001
Yes	894 (21)	406 (24)	488 (19)	
Non–HDL-C, mg/dL				
<190	1504 (90)	617 (89)	887 (90)	.21
≥190	174 (10)	80 (11)	10 (10)	
Lipid-lowering medication use^f^				
No	1742 (95)	708 (94)	1034 (96)	.06
Yes	89 (5)	45 (6)	44 (4)	

Abbreviations: BP, blood pressure; GED, general educational development; HbA_1c_, hemoglobin A_1c_; HDL-C, high-density lipoprotein cholesterol; MGE, male gender expressivity.

SI conversion factors: To convert HbA_1c_ to proportion of total hemoglobin, multiply by 0.01; HDL-C to millimoles per liter, multiply by 0.259.

^a^ *P* values calculated using χ^2^ test with the Rao and Scott second-order correction and Kruskal-Wallis rank-sum test for complex survey samples.

^b^ Included participants who indicated some other race or origin and those who indicated multiple races but did not specify a race.

^c^ Included active-duty military, Department of Veterans Affairs, TRICARE, CHAMPUS, CHAMP VA, or other military health care plan, and Indian Health Services.

^d^ Adult diagnoses were based on responses to survey items in wave V (adulthood) asking, “whether a doctor, nurse, or other healthcare provider ever told you that you have or had [high blood pressure or hypertension, high blood sugar or diabetes, high blood cholesterol, triglycerides, lipids, or hyperlipidemia].”

^e^ Biomeasure outcomes were binary variables based on BP measurements (increased: systolic BP ≥130 mm Hg or diastolic BP ≥80 mm Hg; normal: systolic BP <130 mm Hg and diastolic BP <80 mm Hg) and venous blood samples (increased HbA_1c _≥6.5%; normal HbA_1c_ <6.5%; increased non–HDL-C ≥190 mg/dL; normal non–HDL-C <190 mg/dL).

^f^ Adult medication use was assessed based on self-report of all medications used in 4 weeks before data collection, grouped and categorized into antihypertensive, hypoglycemic, and lipid-lowering agents based on classification in Micromedex and Lexicomp.

**Figure 1.**
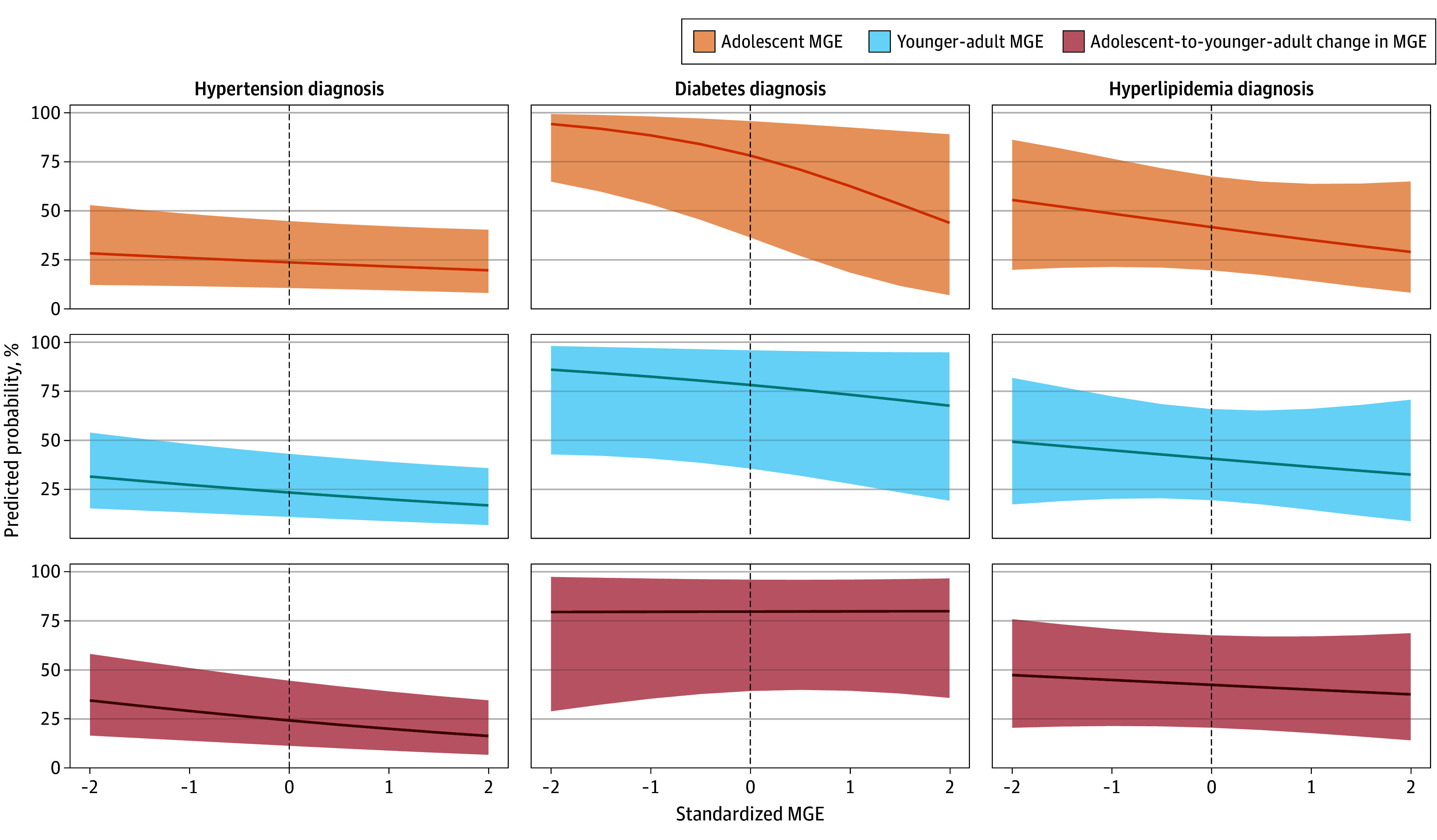
Associations of Male Gender Expressivity (MGE) With Adult Diagnosis of Hypertension, Diabetes, and Hyperlipidemia Higher MGE in adolescence was associated with a lower predicted probability of adult diabetes diagnosis among men with hemoglobin A_1c_ levels greater than or equal to 6.5% (to convert to proportion of total hemoglobin, multiply by 0.01). Higher MGE in younger adulthood and higher adolescent-to-younger-adult MGE changes were associated with lower predicted probabilities of adult hypertension diagnosis among men with increased blood pressure (≥130 mm Hg systolic and/or ≥80 mm Hg diastolic). The curves indicate the average marginal effect of male gender expressivity on each outcome. Shaded areas indicate 95% CIs.

Associations of MGE at each developmental stage with adult CVD risk factor outcomes are reported in Table 2. In model 1 (Figure 1), examining associations of MGE with CVD risk factor diagnoses among participants with biomeasure evidence of these conditions, we found that among adult men with increased BP, every SD increase in younger adult MGE was associated with a 4-percentage point lower probability (MGE, −0.04; 95% CI, −0.07 to −0.01) of adult hypertension diagnoses. Every SD increase in adolescent-to-younger adult MGE change, adjusting for adolescent MGE, was associated with a 5-percentage point (MGE, −0.05; 95% CI, −0.08 to −0.01) lower probability of adult hypertension diagnoses among this group of adult men. Among adult men with HbA_1c_ greater than or equal to 6.5%, every SD increase in adolescent MGE was associated with a 15-percenatge point lower probability (MGE, −0.15; 95% CI, −0.27 to −0.03) of adult diabetes diagnoses. There was no association between MGE and hyperlipidemia diagnoses among men with high non–HDL-C.

**Table 2.** Average Marginal Effects Coefficients Estimating Associations Between MGE and Adult Diagnosis, Treatment, and Biomeasure Evidence of Adult CVD Risks^a^

Model^b^	MGE (95% CI), dy/dx		Change in MGE (95% CI), dy/dx
	Adolescent	Younger adult	
**Model 1 (diagnosis in males with increased biomeasures)** ^c^			
Hypertension	−0.02 (−0.06 to 0.01)	−0.04 (−0.07 to −0.01)^d^	−0.05 (−0.08 to −0.01)^e^
Diabetes	−0.15 (−0.27 to −0.03)^d^	−0.06 (−0.17 to 0.05)	0.00 (−0.11 to 0.11)
Hyperlipidemia	−0.06 (−0.19 to 0.06)	−0.04 (−0.17 to 0.09)	−0.02 (−0.11 to 0.06)
**Model 2 (treatment in males with diagnoses)** ^f^			
Hypertension	−0.11 (−0.16 to −0.06)^e^	−0.07 (−0.13 to −0.01)^d^	−0.02 (−0.08 to 0.03)
Diabetes	−0.05 (−0.18 to 0.08)	−0.10 (−0.20 to −0.01)^d^	−0.09 (−0.18 to 0.01)^g^
Hyperlipidemia	0.01 (−0.05 to 0.06)	0.00 (−0.05 to 0.05)	−0.00 (−0.05 to 0.04)
**Model 3 (biomeasure evidence)** ^h^			
Increased BP	0.00 (−0.03 to 0.03)	−0.02 (−0.06 to 0.01)	−0.02 (−0.06 to 0.01)
Increased HbA_1c_	0.00 (−0.01 to 0.02)	−0.01 (−0.02 to 0.00)	−0.01 (−0.02 to 0.00)
Increased non–HDL-C	0.00 (−0.02 to 0.03)	−0.01 (−0.03 to 0.01)	−0.01 (−0.03 to 0.01)

Abbreviations: BP, blood pressure; CVD, cardiovascular disease; dy/dx, average marginal effects; HbA_1c_, hemoglobin A_1c_; HDL-C, high-density lipoprotein cholesterol; MGE, male gender expressivity.

^a^ Coefficients (dy/dx) are marginal effects coefficients.

^b^ All models were adjusted for race and ethnicity, educational level, insurance status, composite adolescent socioeconomic score, and adolescent neighborhood-level effects.

^c^ Diagnoses were based on yes/no responses to survey items in wave V (adulthood) asking, “whether a doctor, nurse, or other health care provider ever told you that you have or had [high blood pressure or hypertension; high blood sugar or diabetes; high blood cholesterol, triglycerides, lipids, or hyperlipidemia]” in participants with increased biomeasure outcomes, which include binary variables based on BP measurements (increased: systolic BP≥130 mm Hg or diastolic BP≥80 mm Hg; normal: systolic BP<130 mm Hg and diastolic BP<80 mm Hg) and venous blood samples (increased HbA_1c_≥6.5%; normal HbA_1c_<6.5%; increased non–HDL-C≥190 mg/dL; non–HDL-C<190 mg/dL).

^d^ *P* < .05.

^e^ *P* < .01.

^f^ Treatment of CVD risks was based on self-report of all medications used in 4 weeks before data collection, grouped and categorized as antihypertensives, hypoglycemics, and lipid-lowering agents based on classification in Micromedex and Lexicomp in subgroups of participants who self-reported CVD risks.

^g^ *P* < .10.

^h^ Biomeasure evidence of CVD risks based on binary variables based on blood pressure measurements (increased: systolic blood pressure ≥130 mm Hg or diastolic BP≥80 mm Hg; normal: systolic BP<130 and diastolic BP<80 mm Hg) and venous blood samples (increased HbA_1c_≥6.5%; normal HbA_1c_<6.5%; increased non–HDL-C≥190 mg/dL; non–HDL-C<190 mg/dL).

**Figure 2.**
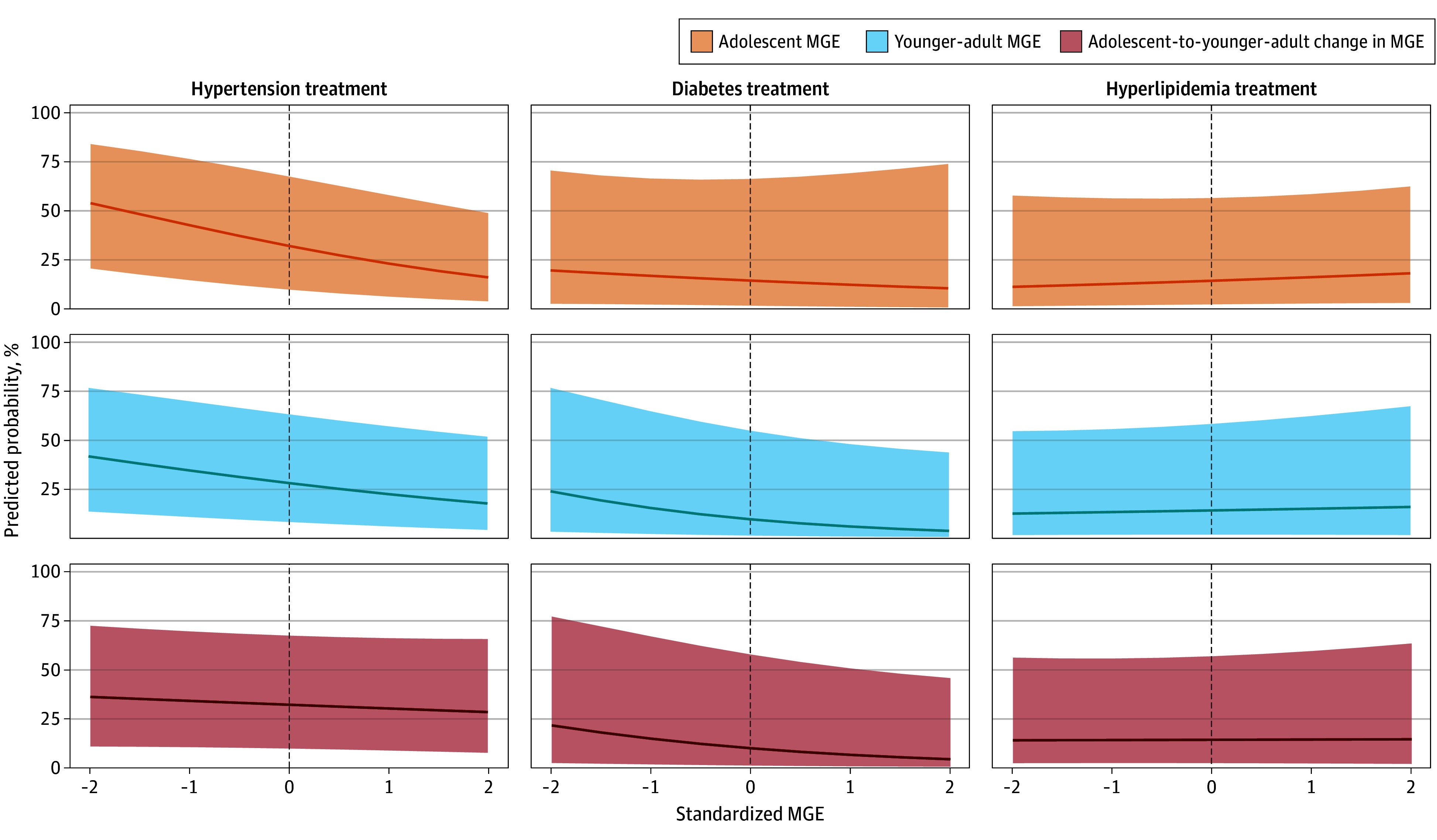
Associations of Male Gender Expressivity (MGE) With Adult Treatment of Hypertension, Diabetes, and Hyperlipidemia Higher MGE in adolescence was associated with a lower predicted probability of adult treatment of hypertension among men who reported hypertension diagnoses. Higher MGE in younger adulthood was associated with a lower predicted probability of adult treatment of hypertension and diabetes among men who reported diagnoses of hypertension and diabetes. The curves indicate the average marginal effect of male gender expressivity on each outcome. Shaded areas indicate 95% CIs.

In model 2 (Figure 2), evaluating associations of MGE with adult treatment, we found that among men who reported hypertension diagnoses, every SD increase in adolescent MGE was associated with an 11-percentage point lower probability (MGE,−0.11; 95% CI, −0.16 to −0.6) of adult antihypertensive use. Each SD increase in younger adult MGE was associated with a 7-percentage point lower probability (MGE, −0.07; 95% CI, −0.13 to −0.01) of adult antihypertensive use. Among adult men who reported diabetes diagnoses, higher younger adult MGE was associated with a lower probability (MGE, −0.10; 95% CI, −0.20 to −0.01) of hypoglycemic use. There was no association between MGE and hyperlipidemia treatment.

In model 3, examining associations of MGE with adult biomeasure outcomes showed no associations of MGE with adult BP, HbA1c, and n-HDL levels. Sensitivity analyses that used a higher BP threshold (systolic BP≥140 mm Hg or diastolic BP≥90 mm Hg) (eTable 4 in Supplement 1) and evaluated associations of MGE with treatment adjusting for biomeasure levels (eTable 5 in Supplement 1) found no substantive differences in observed associations, with 2 exceptions: younger adult MGE was not associated with hypertension diagnoses in men with BPs above the alternate cutoff level or with hypoglycemic use in adults adjusting for HbA_1c_.

## Discussion

In this analysis of nationally representative Add Health data, we found associations between participants’ MGE at multiple developmental stages and downstream modifiable CVD risk diagnoses and treatment, particularly hypertension and diabetes. We did not observe associations of MGE with biomeasure outcomes among adult men aged 32 to 42 years, suggesting similar levels of disease but differences in help-seeking behaviors. Our findings support the hypothesis that prevalent sociocultural pressures to maintain and convey male gender identity—quantified by MGE—may be associated with lower diagnoses and treatment of important, modifiable CVD risks.

Estimates for the prevalence of physiologic CVD risk within our study are consistent with prior reports.^58,68,69,70,71^ For instance, 64% of adult men in our sample displayed hypertensive BP, compared with approximately 59% in published estimates for similar populations,^68^ 5% had diabetes-range HbA_1c_ levels compared with published estimates of approximately 4% in people aged 10 to 44 years,^58,69^ and 10% had increased non–HDL-C levels, also consistent with published estimates.^70,71^ Prior reports have further corroborated that diagnoses of CVD risk conditions frequently do not match higher levels of physiologic risk prevalent in US populations, especially among adolescents and younger adults.^40,41,48^ By some earlier estimates, less than 25% of younger adults with borderline CVD risk levels are aware of their risk.^40^

Such low diagnosis rates are troubling and suggest a need for focused public health messaging. Borderline levels of CVD risk, even in younger adults, have been associated with heightened morbidity and mortality and decreased longevity.^30,31,32,33,34,35,36,37,38,39^ Male gender expressivity was not associated with biomeasure levels in our study, consistent with a lower prevalence during the stage of adulthood examined (age 32-42 years). However, lower levels of diagnosis and treatment remain concerning, given the importance of early recognition and treatment of CVD risk factors.^41,58,59,72,73,74,75^

Prior investigations have explored factors associated with suboptimal CVD prevention efforts.^40,41,48,49,76,77,78^ Such past studies reveal evidence that younger age, lack of insurance, lack of routine care, and belief that one is in excellent health may all be associated with decreased diagnoses.^40,41^ However, to our knowledge, no studies have examined associations with MGE, despite qualitative evidence suggesting that prevalent sociocultural pressures to convey male gender often lead to decreased help-seeking.^27,28,29^

Many efforts to address gender-based disparities in CVD have focused mainly on factors associated with inadequate risk detection and reduction among women.^79,80,81^ Such attempts to improve CVD awareness and treatment among women are necessary. Our findings suggest further efforts are also needed to uncover overlooked mechanisms by which sociocultural pressures around gender may precipitate preventable CVD morbidity and mortality among men.

While our analysis focused on participants who identified as male—without distinguishing between cisgender, transgender, or other gender-diverse identities—prior publications identify manhood generally as a precarious social identity, requiring continuous social proof.^3,4,5,6,82^ Evidence suggests that persons who experience what they believe to be threats to their male gender identity are especially likely to enact compensatory, stereotype-consistent behaviors to reclaim their gender identity, which may include rejecting help.^3,7,27,82,83^ Future efforts should seek to understand how associations of MGE with CVD diagnoses and treatment may be moderated, especially by marginalized social identities, including transgender and other gender-diverse identities. Equally, public health messaging and related efforts to encourage CVD prevention might be designed to reach people for whom interactions of identity and gender expressivity appear predictive of CVD risk.

Our analysis also identified the need for further research regarding how changes in MGE across the life course may relate to downstream CVD risk. We found associations between younger adult MGE and adult hypertension diagnoses. Yet in the case of diabetes, we found associations between adolescent but not younger adult MGE and adult diagnoses. Some of this variation may be due to the mixing of type 1 diabetes, an autoimmune disease that arises mostly in childhood and adolescence, with type 2 diabetes, a form that develops primarily in middle age and older adulthood. We might expect type 1 diabetes recognition and treatment to have less association with MGE, as it is frequently diagnosed in childhood when parents primarily manage health; however, adolescents diagnosed with conditions that undercut their sense of identity (gender or otherwise) may be more likely to downplay their existence.^84,85^ Some variation may also be due to the ways that context-specific, cultural scripts for gender identities change over time, possibly shifting the association between MGE and help-seeking for specific CVD diagnoses across decades.

Relatedly, the lack of an association between MGE and hyperlipidemia diagnoses and treatment provides circumstantial evidence of the potential influence of such context-specific scripts for how male gender is expected to be expressed on specific CVD risk diagnoses and treatment. This lack of significance is consistent with research finding that, unlike hypertension and diabetes, hyperlipidemia control is higher among men than women.^21^ Prior researchers have suggested such disparities may be attributable to sex-linked physiologic differences.^21^ Our results suggest alternative possibilities, including differences in risk-mitigation behavior, possibly associated with direct-to-consumer advertising,^86^ which has been shown to substantially increase statin use.^87^ It is possible that direct-to-consumer advertising messages have shifted cultural beliefs and perceptions associating hyperlipidemia, statin use, and male gender, as with other health behaviors, such as smoking.^1,88,89^

### Limitations

Our findings should be interpreted in the context of several limitations. First, participants’ survey responses may be affected by imperfect recall and social desirability biases, which may co-vary with MGE. Second, Add Health data are unavailable past the fifth decade of life. Since many CVD risk factors are subclinical at younger ages, this may limit our power, biasing findings toward the null. Third, measures of diagnosis may also include findings of increased BP (prehypertension) and blood glucose (prediabetes) below the biomeasure thresholds we used, also potentially biasing findings toward the null. Fourth, BP is ideally based on BP readings over multiple days rather than 3 readings during a single in-home examination. The prevalence of increased BP should be interpreted in this light as well as concerns about a possible white coat effect, although the hypertension prevalence in our study is consistent with earlier population estimates. Add Health BP measurement protocols and materials have also been rigorously validated and reliably assessed.^90,91^ Fifth, restricting some analyses to only those who participated in biomeasure examinations may have introduced bias based on who was likeliest to participate, although this too would have biased results toward the null. Sixth, our measure of treatment (medication use) realistically reflects an interplay between prescribers’ behaviors and participants’ adherence and self-report, and thus cannot be attributed to participants alone.

## Conclusions

The findings of this cohort study suggest important yet easily overlooked connections between prevalent sociocultural pressures to convey masculinity and CVD risk. While we did not find significant links between MGE and biomeasure evidence of CVD risk among adults aged 32 to 42 years, our data revealed associations of MGE with CVD risk diagnoses and treatment. Given what is known about failures to identify and address risks, this finding should inform efforts to improve CVD prevention.

## Notes

Supplement 1. **eFigure.** Survey Participation and Follow-Up **eTable 1.** Survey Items Used to Create MGE Variables in A) Waves I and B) Wave IV **eTable 2.** Item Missingness of Covariates **eTable 3.** Sensitivity Analysis of Missing Data: Comparison of Participants With and Without Complete Data, Add Health Waves I (1994-1995), IV (2008-2009), and V (2016-2018) **eTable 4.** Average Marginal Effects Coefficients *(dy/dx)* Estimating Associations Between MGE and Adult Diagnoses, Treatment, and Biomeasure Evidence of Hypertension Using Alternate Classification **eTable 5.** Marginal Effects Coefficients *(dy/dx)* Estimating Associations Between MGE and Adult Treatment of Modifiable CVD Risks

Supplement 2.
Data Sharing Statement
