# Supplementary material for: Male Gender Expressivity and Diagnosis and Treatment of Cardiovascular Disease Risks in Men
Source: JAMA Netw Open. 2024 Oct 25;7(10):e2441281. doi: 10.1001/jamanetworkopen.2024.41281 (PMC11512345; doi:10.1001/jamanetworkopen.2024.41281)
Supplement: Supplement 1. — eFigure. Survey Participation and Follow-Up eTable 1. Survey Items Used to Create MGE Variables in A) Waves I and B) Wave IV eTable 2. Item Missingness of Covariates eTable 3. Sensitivity Analysis of Missing Data: Comparison of Participants With and Without Complete Data, Add Health Waves I (1994-1995), IV (2008-2009), and V (2016-2018) eTable 4. Average Marginal Effects Coefficients (dy/dx) Estimating Associations Between MGE and Adult Diagnoses, Treatment, and Biomeasure Evidence of Hypertension Using Alternate Classification eTable 5. Marginal Effects Coefficients (dy/dx) Estimating Associations Between MGE and Adult Treatment of Modifiable CVD Risks [file jamanetwopen-e2441281-s001.pdf]

Supplementary Online Content

Glasser NJ, Jameson JC, Huang ES, et al. Male gender expressivity and diagnosis and treatment of cardiovascular disease risks in men. *JAMA Netw Open*. 2024;7(10):e2441281. doi:10.1001/jamanetworkopen.2024.41281

eFigure 1. Survey Participation and Follow-Up

eTable 1. Survey Items Used to Create MGE Variables in A) Waves I and B) Wave IV

eTable 2. Item Missingness of Covariates

eTable 3. Sensitivity Analysis of Missing Data: Comparison of Participants With and Without Complete Data, Add Health Waves I (1994-1995), IV (2008-2009), and V (2016-2018)

eTable 4. Average Marginal Effects Coefficients (dy/dx) Estimating Associations between MGE and Adult Diagnoses, Treatment, and Biomeasure Evidence of Hypertension Using Alternate Classification

eTable 5. Marginal Effects Coefficients (dy/dx) Estimating Associations Between MGE and Adult Treatment of Modifiable CVD Risks

This supplementary material has been provided by the authors to give readers additional information about their work.

© 2024 Glasser NJ et al. *JAMA Network Open.*

eFigure 1. Survey Participation and Follow-Up

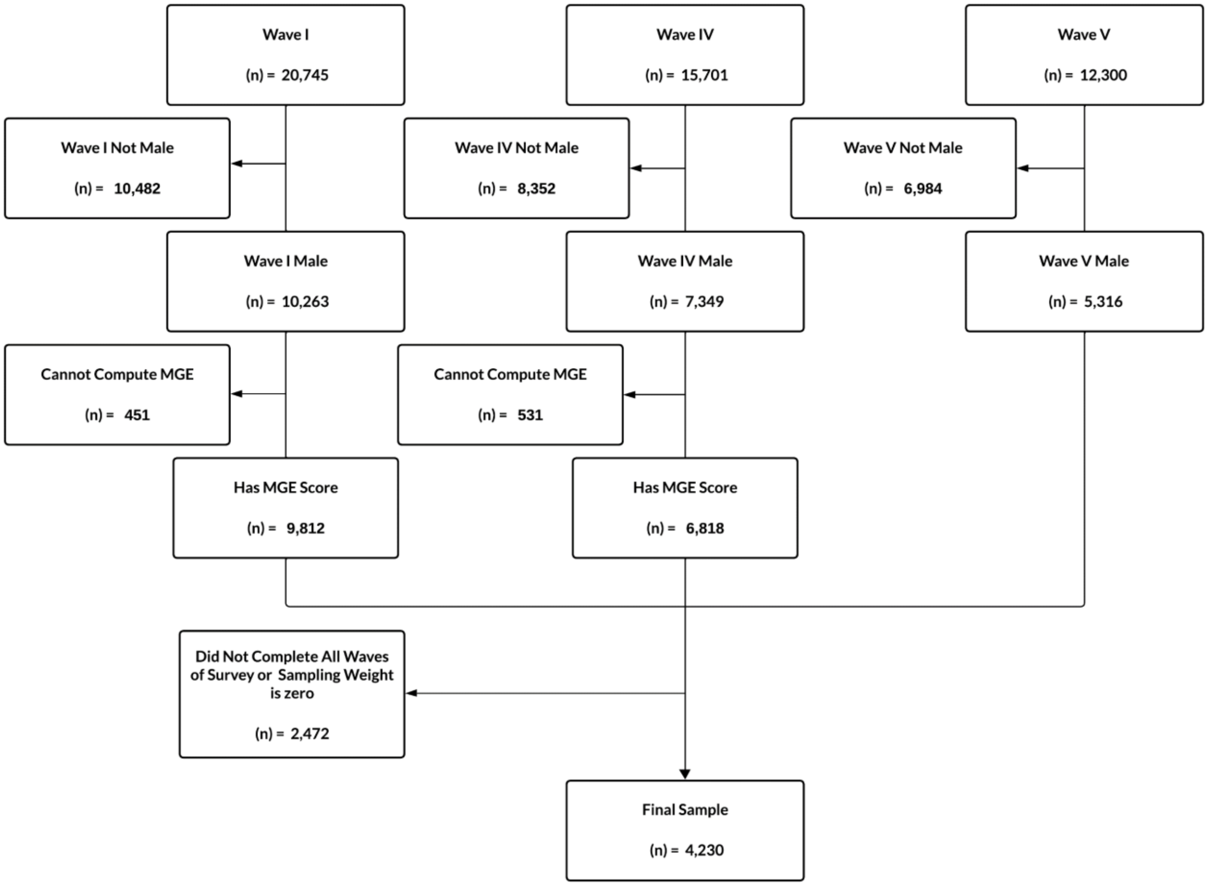

© 2024 Glasser NJ et al. *JAMA Network Open.*

eTable 1. Survey Items used to create MGE variables* in A) Waves I and B) Wave IV

A. Variables used to construct MGE measure Wave I:

Frequency of crying	(0 = Never; 4 = Every day)
Frequency of playing an active sport	(0 = Not at all; 3 = 5 or more times)
Got into a physical fight	(0 = Never; 2 = More than once)
How do you think of yourself in terms of weight?	(1 = Very underweight; 5 = Very overweight)
How much do you feel that your friends care about you?	(1 = Not at all; 5 = Very much)
Hours per week playing video/computer games	(0–99 h)
What do you think your chances are of getting an STD?	(1 = Very high; 5 = No chance)
How many hours do you spend working for pay	(0–140 h)
Have you ever received an out-of-school suspension from school?	(0 = No; 1 = Yes)
Frequency of poor appetite	(0 = Never/Rarely; 3 = Most of the time)
Frequency of wearing a helmet while cycling	(0 = Never; 4 = Always)
Hours per week listening to the radio	(0–99 h)
Frequency of doing work around the house	(0 = Not at all; 3 = 5 or more times)
Upset by difficult problems	(1 = Strongly agree; 5 = Strongly disagree)
How much do you feel adults care about you?	(1 = Not at all; 5 = Very much)
Frequency of moodiness	(0 = Never; 4 = Every day)
You have a lot to be proud of	(1 = Strongly agree; 5 = Strongly disagree)
Have you taken a pledge to remain a virgin until marriage?	(0 = No; 1 = Yes)
Frequency of exercise	(0 = Not at all; 3 = 5 or more times)
Rely on gut feelings to make decisions	(1 = Strongly agree; 5 = Strongly disagree)
Trying to gain/lose/maintain weight?	(1 = Lose; 4 = Nothing)
You never get sad	(1 = Strongly agree; 5 = Strongly disagree)
How likely is it that you will go to college	(1 = Low; 5 = High)
You felt you were just as good as other people	(0 = Never/Rarely; 3 = Most of the time)
Frequency wearing a seatbelt in the car	(0 = Never; 4 = Always)

* Variables identified using a combination of t- and chi-square tests and backward manual regression could be included in a logistic regression model, predicting respondents' "sex" (male or female) with a significance of *p<0.001*

© 2024 Glasser NJ et al. *JAMA Network Open.*

B. Variables used to construct GE measure Wave IV:

Compared to other people your age, how intelligent are you?	(1 = Moderately below; 6 = Extremely above)
I am not interested in other people’s problems	(1 = Strongly agree; 5 = Strongly disagree)
I like to take risks	(1 = Strongly agree; 5 = Strongly disagree)
In the past 24 h, have you participated in vigorous activity long enough to work up a sweat, get your heart thumping, or get out of breath?	(0 = No; 1 = Yes)
I get stressed out easily	(1 = Strongly agree; 5 = Strongly disagree)
I am not really interested in others.	(1 = Strongly agree; 5 = Strongly disagree)
Have you ever used chewing tobacco at least 20 times in your entire life?	(0 = No; 1 = Yes)
Have you ever been arrested?	(0 = No; 1 = Yes)
When you go outside on a sunny day for more than one hour, how likely are you to use sunscreen or sunblock?	(1 = Very likely; 3 = Unlikely)
I have a vivid imagination	(1 = Strongly agree; 5 = Strongly disagree)
I don’t talk a lot	(1 = Strongly agree; 5 = Strongly disagree)
I sympathize with others’ feelings	(1 = Strongly agree; 5 = Strongly disagree)
In the past seven days, how many times did you participate in gymnastics, weightlifting, or strength training?	(0 = Not at all; 7 = 7 or more times)
Hours per week playing video/computer games	(0–105 h)
In past 7 days, how many times did you walk for exercise	(0 = Not at all; 7 = 7 or more times)
I have frequent mood swings	(1 = Strongly agree; 5 = Strongly disagree)
How often do you pray privately?	(0 = Never; 7 = More than once a day)
During typical summer week, how many hours do you spend in the sun during the day?	(0–99 h)
Have you ever been in the military?	(0 = No; 1 = Yes)
In the past 7 days, how many times did you participate in strenuous team sports such as football, soccer, basketball, lacrosse, rugby, field hockey, or ice hockey?	(0 = Not at all; 7 = 7 or more times)
In the past 7 days, you felt too tired to do things.	(0 = Never or rarely; 3 = Most or all of the time)
I worry about things	(1 = Strongly agree; 5 = Strongly disagree

© 2024 Glasser NJ et al. *JAMA Network Open.*

eTable 2. Item Missingness of Covariates

Covariate	% of Participants with Missing Data
Adolescent (Wave I) MGE	3.42%
Young Adult (Wave IV) MGE	5.41%
Race/Ethnicity	0.37%
Educational Attainment	0.09%
Insurance Status	0.93%
Adolescent Socioeconomic Disadvantage	4.65%
Adolescent Neighborhood Disadvantage	0.97%
Missing Any	13.99%

© 2024 Glasser NJ et al. *JAMA Network Open.*

eTable 3. Sensitivity Analysis of Missing Data: Comparison of Participants with and Without Complete Data, Add Health Waves I (1994-1995), IV (2008-2009), and V (2016-2018)

**Sample Characteristics**<br>N = 4,230	Total	Complete cases	Missing cases	p-value[c]
No. (%)	n=4,230	n=	n=1,698	
Race and ethnicity				<0.001
Asian American or Pacific Islander	298 (7)	275 (7)	23 (9)	
Hispanic	487 (12)	442 (11)	45 (18)	
Mixed Race and/or Ethnicity/Others[a]	54 (1)	48 (1)	6 (2)	
Non-Hispanic Black	668 (16)	611 (15)	57 (23)	
Non-Hispanic White	2,711 (64)	2,597 (65)	114 (47)	
Educational Attainment				<0.001
Some High School or Less	168 (4)	150 (4)	18 (7)	
HS Diploma/GED	722 (17)	662 (17)	60 (24)	
Some College or Tech/Assoc. Degree	1730 (41)	1,623 (41)	107 (42)	
College Degree or More	1607 (38)	1,538 (39)	69 (27)	
Insurance Status				<0.001
Private	3,338 (80)	3,183 (80)	155 (69)	
Medicare/Medicaid	308 (7)	278 (7)	30 (13)	
Other Government[b]	162 (4)	154 (4)	8 (4)	
Uninsured	389 (9)	358 (9)	31 (14)	
Adolescent Socioeconomic Disadvantage (mean)	0.51<br>(-4.67, 3.51)	0.51<br>(-4.67, 3.51)	0.21<br>(-3.74, 2.73)	0.07
Adolescent Neighborhood Disadvantage (mean)	23<br>(5, 50)	23<br>(5, 50)	29<br>(7, 50)	<0.001

[a]Included participants who indicated "some other race or origin" and those who indicated multiple races but did not indicate a preferred race.
[b]Included active-duty military, Department of Veterans Affairs, Tri-Care, CHAMPUS, CHAMP VA, or other military health care plan, & Indian Health Services
[c]p-values calculated using chi-squared test with Rao & Scott's second-order correction and Kruskal-Wallis rank-sum test for complex survey samples.

© 2024 Glasser NJ et al. *JAMA Network Open.*

eTable 4. Average Marginal Effects Coefficients (dy/dx) Estimating Associations between MGE and Adult Diagnoses, Treatment, and Biomeasure Evidence of Hypertension Using Alternate Classification^

	Adolescent MGE†	Young adult MGE	Change in MGE
Diagnosis of HTN in Males with elevated BP[a]	-0.00	-0.04	-0.07***
	(-0.07, 0.07)	(-0.10, 0.02)	(-0.12, -0.03)
Treatment of HTN[b]	-0.11***	-0.07***	-0.02
	(-0.16, -0.06)	(-0.13, -0.02)	(-0.08, 0.03)
Elevated Blood Pressure (mm Hg)[c]	-0.00	0.00	0.01
	(-0.03, 0.02)	(-0.02, 0.03)	(-0.02, 0.03)

^Elevated blood pressure defined as systolic blood pressure ≥140 mm Hg or diastolic blood pressure ≥ 90 mm Hg (instead of systolic blood pressure ≥130 mm Hg or diastolic blood pressure ≥ 80 mm Hg as in the main models

†All models adjusted for race/ethnicity, education, insurance status, composite adolescent socio-economic score, and adolescent neighborhood-level effects.

p-value *<0.1, **<0.05, ***<0.01

[a]Diagnoses in males with elevated biomeasures was based on responses to survey items in Wave V (adulthood) asking, "whether a doctor, nurse, or other healthcare provider ever told you that you have or had high blood pressure or hypertension" in subgroups of participants with elevated blood pressure defined as systolic blood pressure ≥140 mm Hg or diastolic blood pressure ≥ 90 mm Hg

[b]Treatment was based on self-report of all antihypertensives used in four weeks prior to data collection, per Micromedex and Lexicomp, in subgroups of participants who self-reported hypertension diagnosis controlling for blood pressure (using the alternate classification [SBP≥140 mm Hg or DBP ≥ 90 mm Hg])

[c]Elevated blood pressure defined as SBP ≥140 mm Hg or diastolic blood pressure ≥ 90 mm Hg

© 2024 Glasser NJ et al. *JAMA Network Open.*

eTable 5. Marginal Effects Coefficients (dy/dx) Estimating Associations between MGE and Adult Treatment of Modifiable CVD Risks[†]

	Adolescent MGE	Young adult MGE	Change in MGE
Model 4[a]			
Treatment of hypertension	-0.11***	-0.07**	-0.02
Treatment of diabetes	-0.04	-0.04**	-0.03
Treatment of hyperlipidemia	0.01	0.00	-0.00

[†]All models adjusted for race/ethnicity, education, insurance status, composite adolescent socio-economic score, and adolescent neighborhood-level effects.
p-value *<0.1, **<0.05, ***<0.01
[a]Treatment of CVD risks was based on self-report of all medications used in four weeks prior to data collection, grouped and categorized into antihypertensives, hypoglycemics, and lipid-lowering agents based on classification in Micromedex and Lexicomp in subgroups of participants who self-reported CVD risks controlling for biomeasure level

© 2024 Glasser NJ et al. *JAMA Network Open.*
