# Supplementary material for: Male Gender Expressivity and Diagnosis and Treatment of Cardiovascular Disease Risks in Men
Source: JAMA Netw Open. 2024 Oct 25;7(10):e2441281. doi: 10.1001/jamanetworkopen.2024.41281 (PMC11512345; doi:10.1001/jamanetworkopen.2024.41281)
Supplement: Supplement 2. — Data Sharing Statement [file jamanetwopen-e2441281-s002.pdf]

Data Sharing Statement

Glasser. Male Gender Expressivity and Diagnosis and Treatment of Cardiovascular Disease Risks in Men. *JAMA Netw Open*. Published October 25, 2024.
doi:10.1001/jamanetworkopen.2024.41281

Data
Data available: Yes
Data types: Deidentified participant data
How to access data: Individual, de-identified participant data that underlie the results reported in this article may be made available through the Carolina Population Center to certified researchers who have a signed data use agreement with the Carolina Population Center. Information is available at the following URL: https://data.cpc.unc.edu/projects/2/view
When available: With publication

Supporting Documents
Document types: Statistical/analytic code
How to access documents: Certified researchers who have a signed data use agreement with the Carolina Population Center, may contact Nathaniel Glasser at nglasser@uchicago.edu or Jacob Jameson at jacobjameson@g.harvard.edu for the statistical/analytic code that underlie the results reported in this article. Statistical/analytic code will also be available at the following URL: https://github.com/jacobjameson/MGE-CVD
When available: With publication

Additional Information
Who can access the data: Certified researchers who have a signed data use agreement with the Carolina Population Center.
Types of analyses: Research intended to produce generalizable knowledge for the purpose of advancing human health.
Mechanisms of data availability: Data will be made available following institutional review board approval and a fully executed data use agreement is in place with the Carolina Population Center.
